# Next generation sequencing yields the complete mitochondrial genome of the darkfin hind *Cephalopholis urodeta* (Serranidae) from the Mischief Reef, South China Sea

**DOI:** 10.1080/23802359.2022.2029602

**Published:** 2022-01-27

**Authors:** Ning Liu, Dianrong Sun, Binbin Shan, Yan Liu, Liangming Wang, Yingbang Huang, Changping Yang

**Affiliations:** a Beijing Aquatic Wildlife Rescue and Conservation Center, Beijing, China; b Key Lab. of South China Sea Fishery Resources Exploration and Utilization, Ministry of Agriculture and Rural Affairs, South China Sea Fisheries Research Institute, Chinese Academy of Fishery Sciences, Guangzhou, China; c Tropical Aquaculture Research and Development Center of South China Sea Fisheries Research Institute, Chinese Academy of Fisheries Sciences, Sanya, China; d Guangdong Provincial Key Laboratory for Healthy and Safe Aquaculture, School of Life Sciences, South China Normal University, Guangzhou, China

**Keywords:** Mitogenome, next generation sequencing, *Cephalopholis urodeta*, Mischief Reef

## Abstract

The complete mitochondrial genome of the *Cephalopholis urodeta* (Serranidae: Epinephelinae) was sequenced by next-generation sequencing method on the basis of one female specimen collected from coral reef areas at the Mischief Reef, South China Sea. The mitogenome is 16,593 bp in length, including 13 protein-coding genes, 22 tRNA genes, two rRNA genes and a D-loop region. The overall content of A + T is 55.66%, which is significantly higher than the C + G content (44.34%). Among four bases, C shows the lowest frequency (15.99%). Phylogenetic tree based on the 13 protein-coding genes was constructed for analyzing the position of *C. urodeta*. The results provide useful insights into further studies on population genetics and phylogenetics of groupers.

As a significant predatory marine fish species, the darkfin hind mainly lives in coral and rocky reefs of the tropical and subtropical oceans worldwide (Heemstra and Randall 1993). At the Mischief Reef, South China Sea, *Cephalopholis urodeta* (Forster, 1801) is one of the dominant fish species on the reef flats and slopes, coexisting with many other groupers, such as *Epinephelus chlorostigma* (Valenciennes, 1828), *E. merra* Bloch, 1793, and *Lutjanus kasmira* (Forsskål, 1775). Though *C. urodeta* is small in body size, it shows important ecological functions in coral-reef ecosystem (Pinault et al. 2014). Several previous studies have been conducted on the stock, taxonomy, evolution, and living habit of *C. urodeta* (Donaldson 2001; Liu and Sadovy 2005; Craig and Hastings 2007; Payet et al. 2016). However, limit effort has been made toward the genetic studies of this species so far. Thus, the present study sequenced the mitochondrial genome of *C. urodeta*, aiming to provide useful molecular markers for further species identification and phylogenetic research.

Samples of *C. urodeta* were collected from the coral reef areas at the Mischief Reef, South China Sea (9°55′N, 115°32′E) in June 2018. A specimen was deposited at the Key Laboratory of South China Sea Fishery Resources Exploration and Utilization, Ministry of Agriculture and Rural Affairs (Changping Yang, yangbing3524@yeah.com) under the voucher number CUF20180601. Total genomic DNA was extracted from the ethanol-preserved muscle tissue using the Marine Animals Genomic DNA Extraction Kit (TIANamp, China). The genomic library construction and next generation sequencing were performed as previously described by Loh et al. (2015). A TruseqTM RNA sample Prep Kit (Illumina Inc., San Diego, CA, USA) was used to construct the whole genome library according to the manufacturer's instructions. The paired-end DNA libraries with insert size of 300–500 bp were PCR-amplified and sequenced on an Illumina HisSeq platform. The clean data without sequencing adapters were then *de novo* assembled by NOVOplasty software. The assembled genes were annotated using the Blast function of NCBI. The locations of the protein-coding genes (PCGs) and the tRNA genes were determined using ORF Finder via NCBI and the MITOS WebServer, respectively (Wang et al. 2018).

The complete mitogenome of *C. urodeta* is 16,593 bp in length (GenBank accession no. MZ411547), containing 37 mitochondrial genes (13 PCGs, 22 tRNAs, and two rRNAs) and a non-coding region. The gene arrangement order is similar to that of previously reported grouper species *C. boenak* (Li et al. 2014). Twenty-eight of the mitochondrial genes are encoded on the heavy strand, while the remaining nine genes are transcribed from the light strand. The overall A + T content is 55.66%, while the C + G content is 44.34%. Similar to the previously reported grouper species (He et al. 2013; Li et al. 2014), cytosine shows the lowest frequency (15.99%) among four bases. Three overlapping reading frames are found among the 13 PCGs, 10 bases between *atp8* and *atp6*, 1 base between *atp6* and *cox3*, and 7 bases between *nad4L* and *nad4*. The start codon of *cox1* and *atp6* is GTG and CTG, respectively, while the remaining 11 PCGs use ATG as start codon. Except for *cox1*, twelve of the 13 PCGs end with TAA terminated codon. The longest gene is *cox1* (1551 bp) in all PCGs, whereas the shortest is *atp8* (168 bp). Twenty-two tRNAs are determined with 68–73 bp in length. One D-loop region (878 bp) is detected between the *trnP* and *trnF* gene. The *rrnL* and *rrnS* gene is 957 bp and 1711 bp in length, respectively. They are located between *trnF* and *trnL*, and are separated by *trnV*.

On the basis of 13 PCGs of 24 fish species, a phylogenetic tree (maximum likelihood method, 1000 bootstrap replicates) was constructed to analyze the position of *C. urodeta*, with the freshwater fish *Eptatretus burger* (Girard, 1855) as out group. The phylogenetic analysis suggested that *C. urodeta* was closely related to *C. boenak* (Bloch, 1790) and *Aethaloperca rogaa* (Forsskål, 1775), rather than the other three grouper species (*Cromileptes altivelis* (Valenciennes, 1828), *Epinephelus areolatus* (Forsskål, 1775), and *E. awoara* (Temminck and Schlegel, 1842) (Figure 1). It can be inferred from the phylogenetic tree that the seven species of the family Serranidae formed good monophyly. This complete mitogenome can provide useful genetic information for future studies on phylogeny and the population of the family Serranidae.

**Figure 1.**
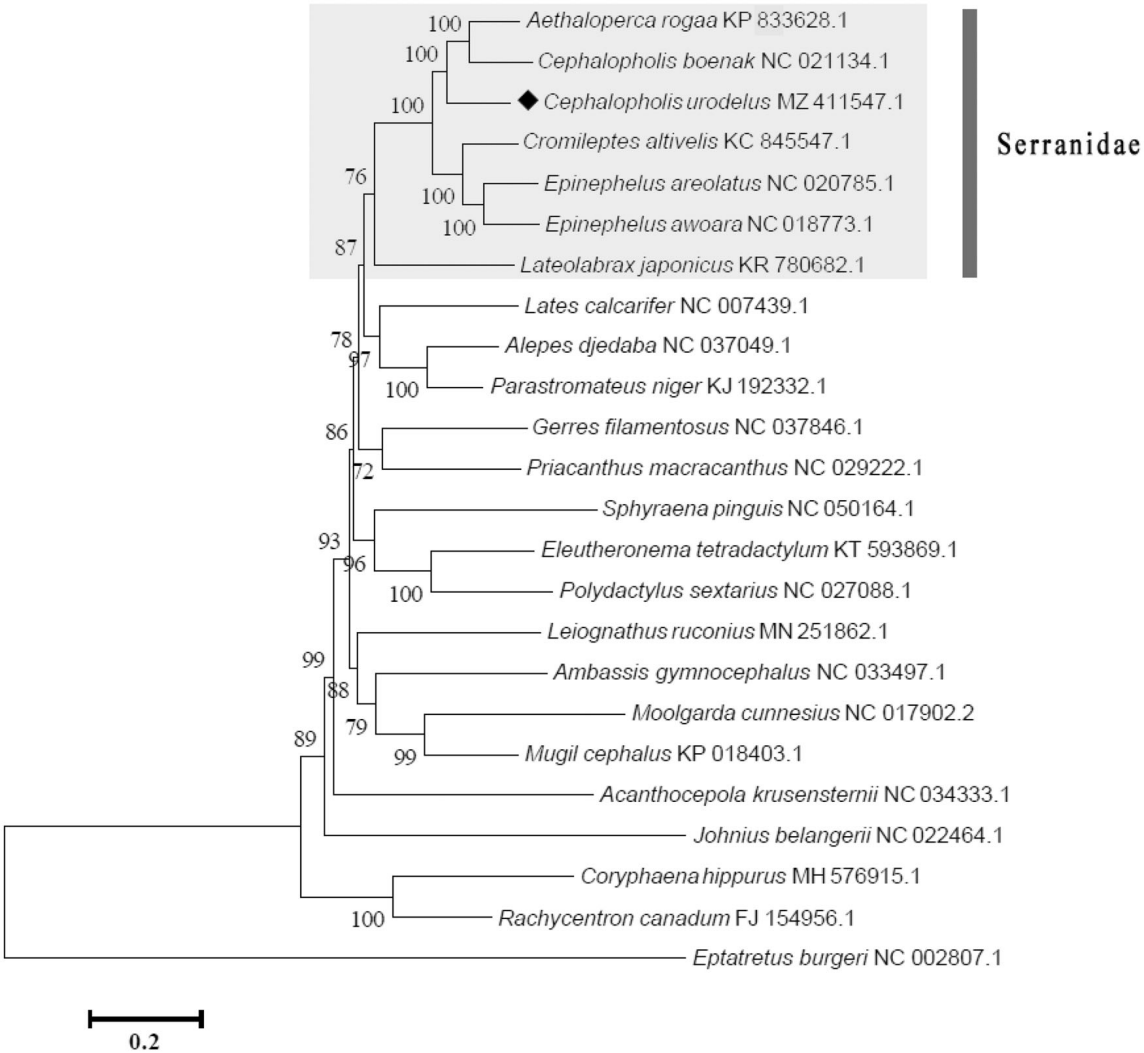
Phylogeny of *Cephalopholis urodeta* based on nucleotide sequences. The phylogenetic tree was inferred from the nucleotide sequences of 13 PCGs using ML method. *Eptatretus burger* was used as outgroup.

## Data Availability

The genome sequence data that support the findings of this study are openly available in GenBank of NCBI at (https://www.ncbi.nlm.nih.gov/) under the accession no. MZ411547. The associated BioProject, SRA, and Bio-Sample numbers are PRJNA758836, SRR15667801, and SAMN21037224, respectively.
